# aenmd: annotating escape from nonsense-mediated decay for transcripts with protein-truncating variants

**DOI:** 10.1093/bioinformatics/btad556

**Published:** 2023-09-09

**Authors:** Jonathan Klonowski, Qianqian Liang, Zeynep Coban-Akdemir, Cecilia Lo, Dennis Kostka

**Affiliations:** Department of Developmental Biology, University of Pittsburgh School of Medicine, Pittsburgh, PA 15201, United States; Department of Developmental Biology, University of Pittsburgh School of Medicine, Pittsburgh, PA 15201, United States; Department of Epidemiology, Human Genetics and Environmental Sciences, University of Texas School of Public Health, Houston, TX 77030, United States; Department of Developmental Biology, University of Pittsburgh School of Medicine, Pittsburgh, PA 15201, United States; Department of Developmental Biology, University of Pittsburgh School of Medicine, Pittsburgh, PA 15201, United States; Department of Computational & Systems Biology and Center for Evolutionary Biology and Medicine, University of Pittsburgh School of Medicine, Pittsburgh, PA 15260,United States

## Abstract

**Summary:**

DNA changes that cause premature termination codons (PTCs) represent a large fraction of clinically relevant pathogenic genomic variation. Typically, PTCs induce transcript degradation by nonsense-mediated mRNA decay (NMD) and render such changes loss-of-function alleles. However, certain PTC-containing transcripts escape NMD and can exert dominant-negative or gain-of-function (DN/GOF) effects. Therefore, systematic identification of human PTC-causing variants and their susceptibility to NMD contributes to the investigation of the role of DN/GOF alleles in human disease. Here we present aenmd, a software for annotating PTC-containing transcript-variant pairs for predicted escape from NMD. aenmd is user-friendly and self-contained. It offers functionality not currently available in other methods and is based on established and experimentally validated rules for NMD escape; the software is designed to work at scale, and to integrate seamlessly with existing analysis workflows. We applied aenmd to variants in the gnomAD, Clinvar, and GWAS catalog databases and report the prevalence of human PTC-causing variants in these databases, and the subset of these variants that could exert DN/GOF effects via NMD escape.

**Availability and implementation:**

aenmd is implemented in the R programming language. Code is available on GitHub as an R-package (github.com/kostkalab/aenmd.git), and as a containerized command-line interface (github.com/kostkalab/aenmd_cli.git).

## 1 Introduction

Nonsense-mediated mRNA decay (NMD) is a well-characterized, evolutionarily conserved quality-control mechanism that is essential for embryogenesis and other developmental processes, and it is known to play a role in human disease (Lykke-Andersen and Jensen 2015). NMD guards against compromised transcripts by affecting their degradation versus translation, including transcripts with variants that introduce premature termination codons (PTCs). PTC-causing variants where a resulting transcript is subject to NMD can exert loss-of-function (LOF) effects in case of haploinsufficiency, where transcripts from both chromosomes are required for normal protein function. For PTC-harboring transcripts that escape NMD, there are additional possibilities of dominant-negative (DN) or gain-of-function (GOF) effects, where the altered protein may interfere with the wild-type version (DN) or where it can possess an altered molecular function or activity domain (GOF). While molecular mechanisms of DN/GOF effects are generally less well understood compared with LOF effects (Backwell and Marsh 2022) and the pathogenicity of NMD-escaping variants is gene/transcript-specific, they do play a significant role in human disease (Cheng *et al.* 1994, Inoue *et al.* 2004, 2007, Khajavi *et al.* 2006, Inácio *et al.* 2007, Bhuvanagiri *et al.* 2010, Miller and Pearce 2014, Lykke-Andersen and Jensen 2015, Coban-Akdemir *et al.* 2018, Miyake *et al.* 2020, Gerasimavicius *et al.* 2022).

Given the potential contribution of PTC variants with NMD escape in causing disease, significant new insights into mechanisms of disease pathogenicity can emerge from annotating PTC-containing transcripts with a prediction about their escape from NMD.(Hall and Thein 1994, Frischmeyer and Dietz 1999, Kerr *et al.* 2001, Inácio *et al.* 2004, 2007, Inoue *et al.* 2004, 2007, Khajavi *et al.* 2006, Mort *et al.* 2008, Bhuvanagiri *et al.* 2010, Miller and Pearce 2014, White *et al.* 2015, 2016, Bayram *et al.* 2017, Jansen *et al.* 2017, Poli *et al.* 2018, Hamanaka *et al.* 2020, Miyake *et al.* 2020, Supek *et al.* 2021, Kim *et al.* 2022). Using an exon–exon junction complex-dependent model of NMD, a notable fraction of PTC-harboring transcripts is predicted to escape NMD (Coban-Akdemir *et al.* 2018). However, this model only describes about half of NMD-escaping human variation accurately, prompting the development of additional approaches for predicting transcript escape from NMD (Nagy and Maquat 1998, Le Hir *et al.* 2000, 2001, Ishigaki *et al.* 2001, Kim *et al.* 2001, Lykke-Andersen *et al.* 2001, Gehring *et al.* 2003, Inácio *et al.* 2004, Silva *et al.* 2006, Lappalainen *et al.* 2013, Schweingruber *et al.* 2013, Lykke-Andersen and Jensen 2015, Rivas *et al.* 2015, Lindeboom *et al.* 2016, 2019, Hoek *et al.* 2019, Nogueira *et al.* 2021, Supek *et al.* 2021). Nevertheless, and despite the relevance of annotating PTC-causing variants with respect to a modified transcript’s susceptibility to NMD, there is a lack of scalable and accessible software addressing that task comprehensively (i.e. for all types of PTC-causing variants). Therefore, we developed aenmd — a software tool for comprehensive annotation of PTC-causing variant-transcript pairs with (predicted) escape from NMD. aenmd makes use of well-established and experimentally validated rules based on PTC location within a transcript’s intron–exon structure (Lindeboom *et al.* 2016, 2019), and it integrates well into existing variant analysis pipelines. In the following, we describe aenmd in more detail and report statistics of NMD escape for PTC-causing variants in the Clinvar (Landrum *et al.* 2018), gnomAD (Karczewski *et al.* 2020), and NHGRI-EBI GWAS catalog (Sollis *et al.* 2023) resources.

## 2 Materials and methods

### 2.1 Annotating escape from NMD

aenmd predicts escape from NMD for combinations of transcripts and PTC-generating variants by applying a set of NMD escape rules, which are based on where the PTC is located within the mutant transcript. First, the location of the 5′-most (novel) PTC is determined, and then escape from NMD is predicted by the following five rules (Lindeboom *et al.* 2016, 2019): Whether

- the PTC located in the last coding exon (last exon rule),
- the PTC located within d_pen bp upstream of the penultimate exon boundary (penultimate exon rule; default: d_pen = 50)
- the PTC located within d_css bp downstream of the coding start site (css rule default: d_css = 150)
- the PTC located within an exon spanning more than 407bp (407 bp rule)
- the transcript is intronless (single exon rule)

**Figure 1.**
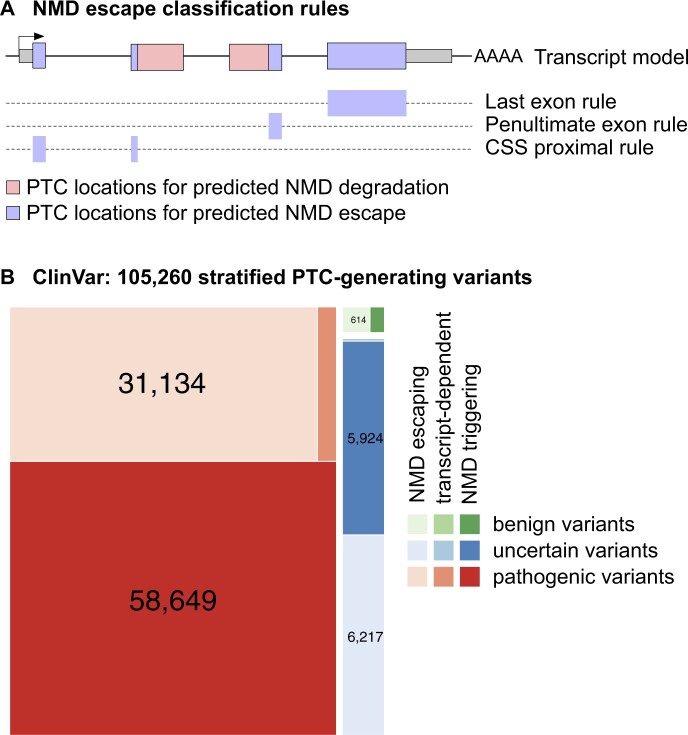
(A) Rules for predicting escape from NMD, with purple-shaded regions indicating areas that would harbor predicted NMD-escaping PTCs. The single exon rule and the 407 bp rule are not shown. (B) ClinVar variants, stratified by pathogenicity and annotated with predicted escape from NMD. transcript-dependent: the same variant overlaps multiple Transcripts and has differing NMD escape predictions.

See Fig. 1A. Distances (in bp) are calculated using the PTC nucleotide closest to the coding start site (CSS) or exon boundary for the css and penultimate exon rules, respectively; variants are assumed to be left-normalized (Tan *et al.* 2015) (aenmd provides this functionality). Variants that overlap exon–intron boundaries or splice regions are not currently analyzed by aenmd. Variant-transcript pairs with a PTC conforming to any of the above rules will be annotated to escape NMD, but results for all rules are reported individually by aenmd; this allows users to focus on subsets of rules, if desired. aenmd is implemented in the R programming language (www.r-project.org), making use of the VariantAnnotation (Obenchain *et al.* 2014) and vcfR (Knaus and Grunwald 2017) packages for importing/exporting variants from/into variant call format (VCF) files, and the Biostrings (Lifschitz *et al.* 2022) and GenomicRanges (Lawrence *et al.* 2013) packages for calculating rules. An index containing all PTC-generating SNVs is pre-calculated for a given transcript set and stored in a trie data structure for lookup, using the triebeard package. For non-SNV variants, alternative alleles for overlapping transcripts are explicitly constructed and assessed. This strategy allows us to assess frameshift variants where a PTC is produced downstream of the variant location, and it accounts for both the size and content of sequence insertions, deletions, and insertion–deletions.

### 2.2 Data on genetic variants and transcript models

We obtained gnomAD version v2.1.11liftover GRCh38, Clinvar version 20221211, and the NHGRI-EBI GWAS version 20220730, catalog from their respective download sites and annotated variants using aenmd. For our analyses, we used transcript models from ENCODE version 105, where we focused on protein-coding transcripts on standard chromosomes that: (i) have an annotated transcript support level of one (or NA for single exon transcripts), and (ii) have a coding sequence length divisible by three.

## 3 Results

### 3.1 aenmd data package

The aenmd R-package provides functionality to annotate variant-transcript pairs for predicted escape from NMD within the R ecosystem. Data dependencies (i.e. transcript models) are implemented via specific data packages (see below), and functionality for data import and export (VCF files) is also provided, as is functionality for variant left-normalization. Key differences that set aenmd apart from currently available tools for annotating escape from NMD are: all types of PTC-causing variants (including frameshift variants that do not cause stop codons at the variant site) are annotated, variants are annotated at scale, inserted sequence is considered for indels, and differentiated (i.e. rule-specific) output is provided for each transcript-variant pair where NMD escape rules are applicable. This enables users to focus on the subset of rules most applicable to their situation; for example, some users may choose to focus on the exon–exon junction complex related “canonical” NMD rules only and ignore the “css proximal,” “single exon,” and “407 bp plus” rules (see Section 2). In addition to the R-package, we also provide a command-line interface to aenmd’s functionality.

### 3.2 aenmd_cli command-line interface

We constructed a containerized version of aenmd with all dependencies, which also provides a command-line interface. This allows end-to-end annotation of variants. An input VCF file is read, PTC-generating variants that overlap a specific transcript set (see the aenmd data packages section below) are annotated, and the annotation results are then included in the INFO column of an output VCF file. In this way, the aenmd_cli command-line tool makes aenmd easily accessible and its results reproducible; there are no external dependencies, no knowledge of the R programming language is required, and it can be seamlessly integrated into existing variant processing workflows.

### 3.3 aenmd data packages

Annotation for (predicted) escape from NMD is based on the location of a PTC in the context of a transcript model. With aenmd, we provide precompiled annotation packages that provide comprehensive protein-coding transcript sets for the GRCh37 and GRCh38 assemblies of the human genome (data packages: aenmd.data.gencode.v43 and aenmd.data.gencode.v43.grch37, respectively), based on GENCODE version 43 annotations. We also provide a more stringently filtered transcript set based on ENSEMBL (version 105), containing transcripts with the highest level of transcript support (data package: aenmd.data.ensdb.v105). The aenmd package provides functionality to select between different transcript sets, allowing convenient prediction of NMD escape for GRCh37 and GRCh38 variants.

### 3.4 Annotation of gnomAD, Clinvar, and the GWAS catalog

We used aenmd with a high-quality ENSEMBL transcript set (aenmd.data.ensdb.v105 annotation package, see above) to annotate the gnomAD, Clinvar, and GWAS catalog databases of human genetic variation for PTC-generating variants predicted to escape NMD. Our results are summarized in Supplementary Tables S1–S3. We observe that the fraction of NMD escape PTC-generating variants varies between 36% (ClinVar), 50% (gnomAD), and 57% (GWAS catalog). The fraction of coding variants in each database that introduce PTCs also varies (10% for ClinVar, 4.1% for gnomAD, and 4.5% for the GWAS catalog). While the absolute number of PTC-generating variants is low for the GWAS catalog (most of its variants are non-coding), we learn from gnomAD that half of the ∼300k PTC-generating variants recovered from ∼125k exome sequences are predicted to escape NMD. Analyzing the ClinVar database (Fig. 1B, Supplementary Table S2), we find that for the subset of variants that are considered pathogenic and generate PTCs, 34% (∼31k variants) are predicted to escape NMD. This suggests that escape from NMD may play a substantial role in the disease mechanisms underlying variants of clinical significance annotated in ClinVar.

### 3.5 Comparison with VEP NMD plugin

We note that Ensemble’s Variant Effect Predictor (VEP, Hunt *et al.* 2022) provides an NMD annotation plugin that annotates escape from NMD for “stop_gained” variants. This set of variants does not include frameshift variants with a downstream PTC, so the set of variants considered by aenmd and the VEP plugin are inherently different. For example, aenmd annotates ∼200k variant-transcript pairs for ClinVar, while VEP considers ∼77k due to its restrictions on variant type (Supplementary Table S4).

Nevertheless, we systematically compared VEP and aenmd NMD escape predictions for the ClinVar database for variants that overlap in transcript set and variant type between the two methods. Overall, we find high consistency of NMD escape predictions (97.5% identical predictions), with 773 (out of 75 840) variant-transcript pairs annotated as NMD-escaping by aenmd but not VEP, and with 1096 pairs annotated as NMD escaping by VEP but not aenmd. We manually examined a limited set of 20 randomly selected variants with different predictions; results are summarized in Supplementary Table S5, and differences are often due to understandable technical differences in the implementation of NMD escape rules.

## 4 Discussion

Here we present aenmd, a self-contained, accessible, and scalable computational tool for annotating (predicted) escape from NMD for variants that generate premature termination codons (PTCs) in a transcript.

While there exist other tools that annotate escape from NMD for PTC variants, aenmd is unique in its specific features. For instance, VEP annotates NMD escape but is limited to “stop_gained” variants. Additionally, a user is unable to readily interpret which NMD escape rules underlie a certain prediction. This interpretability shortcoming is shared with NMDetective (Lindeboom *et al.* 2019), a tool that annotates annotate escape from NMD and provides a NMD efficacy prediction, a feature that aenmd lacks; however, NMDetective’s approach does not consider inserted sequence for indels that cause PTCs. The latter is also the case for the tool NMDescPredictor (Coban-Akdemir *et al.* 2018), which is also different from aenmd in that it does not provide batch annotation functionality, and it implements a smaller set of NMD escape rules. The tool ALoFT (Balasubramanian *et al.* 2017) more generally predicts pathogenicity of loss-of-function variants, but it has the capability to annotate NMD escape. However, its output is less fine-grained than aenmd’s as to which specific rules drive NMD escape annotations. Similarly, the variant annotator SNPEff (Cingolani *et al.* 2012) provides NMD escape prediction, but it only considers two NMD escape rules (penultimate and last exon rules). In summary, aenmd stands out in terms of its functionality, flexibility, and interpretability of results.

We also performed a detailed comparison of aenmd with the VEP NMD plugin, which annotates fewer variant types, uses a smaller set of NMD escape rules, and does not report the outcome of individual rules. While aenmd annotates substantially more variants, we nevertheless found that overlapping predictions were highly consistent.

We note that the rules aenmd (and other tools) utilize for predicting escape from NMD do not yield perfect annotations, and not all the rules are believed to work equally well. For instance, Lindeboom *et al.* (2019) in their NMDetective-B model observe the highest efficacy for the “last exon” rule, followed by the “CSS proximal” rule, followed by the “penultimate exon” rule, followed by the “407bp plus” rule when analyzing cancer data. Further on, it is conceivable that the efficacy of different rules changes across different tissues/cell-types where affected transcripts are expressed. However, a recent study leveraging GTEx data (Teran *et al.* 2021) found that NMD effects were highly stable across tissues and individuals, and it concludes that NMD prediction tools’ predictive power should be stable across tissues.

In addition, we note that NMD plays a role in designing CRISPR gene editing experiments (Lindeboom *et al.* 2019), and therefore aenmd’s functionality will potentially be useful in this context as well.

In summary, aenmd’s comprehensive features, flexibility, and ease of use allow for improved annotation of PTC-generating variants at low computational cost.

## Supplementary Material

btad556_Supplementary_Data

## Data Availability

gnomAD v2.1.1 liftover was downloaded from gnomAD’s downloads website: https://gnomad.broadinstitute.org/downloads. The Clinvar dataset download, version 2022-12-11 from https://ftp.ncbi.nlm.nih.gov/pub/clinvar/vcf_GRCh38/archive_2.0/2022/. NHGRI-EBI Catalog of human genome-wide association studies, version 2022-07-30, was downloaded from https://www.ebi.ac.uk/gwas/docs/file-downloads. Annotation packages for aenmd are available on GitHub (github.com/kostkalab/aenmd_data.git). The source code used to generate results in this manuscript is available on GitHub (github.com/kostkalab/aenmd_manuscript.git).

## References

[btad556-B1] Backwell L , Marsh JA. Diverse molecular mechanisms underlying pathogenic protein mutations: beyond the loss-of-function paradigm. Annu Rev Genomics Hum Genet 2022;23:475–98. 35395171 10.1146/annurev-genom-111221-103208

[btad556-B2] Balasubramanian S , Fu Y, Pawashe M et al Using ALoFT to determine the impact of putative loss-of-function variants in protein-coding genes. Nat Commun 2017;8:382. 28851873 10.1038/s41467-017-00443-5 PMC5575292

[btad556-B3] Bayram Y , White JJ, Elcioglu N et al; Baylor-Hopkins Center for Mendelian Genomics. REST final-exon-truncating mutations cause hereditary gingival fibromatosis. Am J Hum Genet 2017;101:149–56. 28686854 10.1016/j.ajhg.2017.06.006 PMC5501868

[btad556-B4] Bhuvanagiri M , Schlitter AM, Hentze MW et al NMD: RNA biology meets human genetic medicine. Biochem J 2010;430:365–77. 20795950 10.1042/BJ20100699

[btad556-B5] Cheng J , Belgrader P, Zhou X et al Introns are cis effectors of the nonsense-codon-mediated reduction in nuclear mRNA abundance. Mol Cell Biol 1994;14:6317–25. 8065363 10.1128/mcb.14.9.6317-6325.1994 PMC359158

[btad556-B6] Cingolani P , Platts A, Wang LL et al A program for annotating and predicting the effects of single nucleotide polymorphisms, SnpEff: SNPs in the genome of Drosophila melanogaster strain w1118; iso-2; iso-3. Fly (Austin) 2012;6:80–92. 22728672 10.4161/fly.19695 PMC3679285

[btad556-B7] Coban-Akdemir Z , White JJ, Song X et al; Baylor-Hopkins Center for Mendelian Genomics. Identifying genes whose mutant transcripts cause dominant disease traits by potential gain-of-function alleles. Am J Hum Genet 2018;103:171–87. 30032986 10.1016/j.ajhg.2018.06.009 PMC6081281

[btad556-B8] Frischmeyer PA , Dietz HC. Nonsense-mediated mRNA decay in health and disease. Hum Mol Genet 1999;8:1893–900. 10469842 10.1093/hmg/8.10.1893

[btad556-B9] Gehring NH , Neu-Yilik G, Schell T et al Y14 and hUpf3b form an NMD-activating complex. Mol Cell 2003;11:939–49. 12718880 10.1016/s1097-2765(03)00142-4

[btad556-B10] Gerasimavicius L , Livesey BJ, Marsh JA. Loss-of-function, gain-of-function and dominant-negative mutations have profoundly different effects on protein structure. Nat Commun 2022;13:3895. 35794153 10.1038/s41467-022-31686-6 PMC9259657

[btad556-B11] Hall GW , Thein S. Nonsense codon mutations in the terminal exon of the beta-globin gene are not associated with a reduction in beta-mRNA accumulation: a mechanism for the phenotype of dominant beta-thalassemia. Blood 1994;83:2031–7. 8161774

[btad556-B12] Hamanaka K , Imagawa E, Koshimizu E et al De novo truncating variants in the last exon of SEMA6B cause progressive myoclonic epilepsy. Am J Hum Genet 2020;106:549–58. 32169168 10.1016/j.ajhg.2020.02.011 PMC7118575

[btad556-B13] Hoek TA , Khuperkar D, Lindeboom RGH et al Single-molecule imaging uncovers rules governing nonsense-mediated mRNA decay. Mol Cell 2019;75:324–339.e11. 31155380 10.1016/j.molcel.2019.05.008 PMC6675935

[btad556-B14] Hunt SE , Moore B, Amode RM et al Annotating and prioritizing genomic variants using the Ensembl Variant Effect Predictor - a tutorial. Hum Mutat 2022;43:986–97. 34816521 10.1002/humu.24298 PMC7613081

[btad556-B15] Inácio Â , Silva AL, Morgado A et al Comment on ‘Nonsense-mediated mRNA decay modulates clinical outcome of genetic disease’. Eur J Hum Genet 2007;15:533–4; author reply 534. 17342150 10.1038/sj.ejhg.5201808

[btad556-B16] Inácio Â , Silva AL, Pinto J et al Nonsense mutations in close proximity to the initiation codon fail to trigger full nonsense-mediated mRNA decay. J Biol Chem 2004;279:32170–80. 15161914 10.1074/jbc.M405024200

[btad556-B17] Inoue K , Khajavi M, Ohyama T et al Molecular mechanism for distinct neurological phenotypes conveyed by allelic truncating mutations. Nat Genet 2004;36:361–9. 15004559 10.1038/ng1322

[btad556-B18] Inoue K , Ohyama T, Sakuragi Y et al Translation of SOX10 3' untranslated region causes a complex severe neurocristopathy by generation of a deleterious functional domain. Hum Mol Genet 2007;16:3037–46. 17855451 10.1093/hmg/ddm262

[btad556-B19] Ishigaki Y , Li X, Serin G et al Evidence for a pioneer round of mRNA translation: mRNAs subject to nonsense-mediated decay in mammalian cells are bound by CBP80 and CBP20. Cell 2001;106:607–17. 11551508 10.1016/s0092-8674(01)00475-5

[btad556-B20] Jansen S , Geuer S, Pfundt R et al; Deciphering Developmental Disorders Study. De novo truncating mutations in the last and penultimate exons of PPM1D cause an intellectual disability syndrome. Am J Hum Genet 2017;100:650–8. 28343630 10.1016/j.ajhg.2017.02.005 PMC5384016

[btad556-B21] Karczewski KJ , Francioli LC, Tiao G et al; Genome Aggregation Database Consortium. The mutational constraint spectrum quantified from variation in 141,456 humans. Nature 2020;581:434–43. 32461654 10.1038/s41586-020-2308-7 PMC7334197

[btad556-B22] Kerr TP , Sewry CA, Robb SA et al Long mutant dystrophins and variable phenotypes: evasion of nonsense-mediated decay? Hum Genet 2001;109:402–7. 11702221 10.1007/s004390100598

[btad556-B23] Khajavi M , Inoue K, Lupski JR. Nonsense-mediated mRNA decay modulates clinical outcome of genetic disease. Eur J Hum Genet 2006;14:1074–81. 16757948 10.1038/sj.ejhg.5201649

[btad556-B24] Kim HJ , Mohassel P, Donkervoort S et al Heterozygous frameshift variants in HNRNPA2B1 cause early-onset oculopharyngeal muscular dystrophy. Nat Commun 2022;13:2306. 35484142 10.1038/s41467-022-30015-1 PMC9050844

[btad556-B25] Kim VN , Kataoka N, Dreyfuss G. Role of the nonsense-mediated decay factor hUpf3 in the splicing-dependent exon-exon junction complex. Science 2001;293:1832–6. 11546873 10.1126/science.1062829

[btad556-B26] Knaus BJ , Grunwald NJ. vcfr: a package to manipulate and visualize variant call format data in R. Mol Ecol Resour 2017;17:44–53. 27401132 10.1111/1755-0998.12549

[btad556-B27] Landrum MJ , Lee JM, Benson M et al ClinVar: improving access to variant interpretations and supporting evidence. Nucleic Acids Res 2018;46:D1062–7. 29165669 10.1093/nar/gkx1153 PMC5753237

[btad556-B28] Lappalainen T , Sammeth M, Friedländer MR et al; Geuvadis Consortium. Transcriptome and genome sequencing uncovers functional variation in humans. Nature 2013;501:506–11. 24037378 10.1038/nature12531 PMC3918453

[btad556-B29] Lawrence M , Huber W, Pagès H et al Software for computing and annotating genomic ranges. PLoS Comput Biol 2013;9:e1003118. 23950696 10.1371/journal.pcbi.1003118 PMC3738458

[btad556-B30] Le Hir H , Gatfield D, Izaurralde E et al The exon-exon junction complex provides a binding platform for factors involved in mRNA export and nonsense-mediated mRNA decay. EMBO J 2001;20:4987–97. 11532962 10.1093/emboj/20.17.4987 PMC125616

[btad556-B31] Le Hir H , Izaurralde E, Maquat LE et al The spliceosome deposits multiple proteins 20-24 nucleotides upstream of mRNA exon-exon junctions. EMBO J 2000;19:6860–9. 11118221 10.1093/emboj/19.24.6860 PMC305905

[btad556-B32] Lifschitz S , Haeusler EH, Catanho M et al Bio-strings: a relational database data-type for dealing with large biosequences. BioTech (Basel) 2022;11;31. 35997339 10.3390/biotech11030031 PMC9472027

[btad556-B33] Lindeboom RGH , Supek F, Lehner B. The rules and impact of nonsense-mediated mRNA decay in human cancers. Nat Genet 2016;48:1112–8. 27618451 10.1038/ng.3664 PMC5045715

[btad556-B34] Lindeboom RGH , Vermeulen M, Lehner B et al The impact of nonsense-mediated mRNA decay on genetic disease, gene editing and cancer immunotherapy. Nat Genet 2019;51:1645–51. 31659324 10.1038/s41588-019-0517-5 PMC6858879

[btad556-B35] Lykke-Andersen J , Shu MD, Steitz JA. Communication of the position of exon-exon junctions to the mRNA surveillance machinery by the protein RNPS1. Science 2001;293:1836–9. 11546874 10.1126/science.1062786

[btad556-B36] Lykke-Andersen S , Jensen TH. Nonsense-mediated mRNA decay: an intricate machinery that shapes transcriptomes. Nat Rev Mol Cell Biol 2015;16:665–77. 26397022 10.1038/nrm4063

[btad556-B37] Miller JN , Pearce DA. Nonsense-mediated decay in genetic disease: friend or foe? Mutat Res Rev Mutat Res 2014;762:52–64. 25485595 10.1016/j.mrrev.2014.05.001 PMC4260155

[btad556-B38] Miyake N , Takahashi H, Nakamura K et al Gain-of-function MN1 truncation variants cause a recognizable syndrome with craniofacial and brain abnormalities. Am J Hum Genet 2020;106:13–25. 31839203 10.1016/j.ajhg.2019.11.011 PMC7042485

[btad556-B39] Mort M , Ivanov D, Cooper DN et al A meta-analysis of nonsense mutations causing human genetic disease. Hum Mutat 2008;29:1037–47. 18454449 10.1002/humu.20763

[btad556-B40] Nagy E , Maquat LE. A rule for termination-codon position within intron-containing genes: when nonsense affects RNA abundance. Trends Biochem Sci 1998;23:198–9. 9644970 10.1016/s0968-0004(98)01208-0

[btad556-B41] Nogueira G , Fernandes R, García-Moreno JF et al Nonsense-mediated RNA decay and its bipolar function in cancer. Mol Cancer 2021;20:72. 33926465 10.1186/s12943-021-01364-0 PMC8082775

[btad556-B42] Obenchain V , Lawrence M, Carey V et al VariantAnnotation: a Bioconductor package for exploration and annotation of genetic variants. Bioinformatics 2014;30:2076–8. 24681907 10.1093/bioinformatics/btu168 PMC4080743

[btad556-B43] Poli MC , Ebstein F, Nicholas SK et al; Undiagnosed Diseases Network members. Heterozygous truncating variants in POMP escape Nonsense-Mediated decay and cause a unique immune dysregulatory syndrome. Am J Hum Genet 2018;102:1126–42. 29805043 10.1016/j.ajhg.2018.04.010 PMC5992134

[btad556-B44] Rivas MA , Pirinen M, Conrad DF et al; Geuvadis Consortium. Human genomics. Effect of predicted protein-truncating genetic variants on the human transcriptome. Science 2015;348:666–9. 25954003 10.1126/science.1261877 PMC4537935

[btad556-B45] Schweingruber C , Rufener SC, Zünd D et al Nonsense-mediated mRNA decay - mechanisms of substrate mRNA recognition and degradation in mammalian cells. Biochim Biophys Acta 2013;1829:612–23. 23435113 10.1016/j.bbagrm.2013.02.005

[btad556-B46] Silva AL , Pereira FJC, Morgado A et al The canonical UPF1-dependent nonsense-mediated mRNA decay is inhibited in transcripts carrying a short open reading frame independent of sequence context. RNA 2006;12:2160–70. 17077274 10.1261/rna.201406 PMC1664719

[btad556-B47] Sollis E , Mosaku A, Abid A et al The NHGRI-EBI GWAS catalog: knowledgebase and deposition resource. Nucleic Acids Res 2023;51:D977–85. 36350656 10.1093/nar/gkac1010 PMC9825413

[btad556-B48] Supek F , Lehner B, Lindeboom RGH. To NMD or not to NMD: nonsense-mediated mRNA decay in cancer and other genetic diseases. Trends Genet 2021;37:657–68. 33277042 10.1016/j.tig.2020.11.002

[btad556-B49] Tan A , Abecasis GR, Kang HM. Unified representation of genetic variants. Bioinformatics 2015;31:2202–4. 25701572 10.1093/bioinformatics/btv112 PMC4481842

[btad556-B50] Teran NA , Nachun DC, Eulalio T et al Nonsense-mediated decay is highly stable across individuals and tissues. Am J Hum Genet 2021;108:1401–8. 34216550 10.1016/j.ajhg.2021.06.008 PMC8387471

[btad556-B51] White J , Mazzeu JF, Hoischen A et al; Baylor-Hopkins Center for Mendelian Genomics. DVL1 frameshift mutations clustering in the penultimate exon cause autosomal-dominant robinow syndrome. Am J Hum Genet 2015;96:612–22. 25817016 10.1016/j.ajhg.2015.02.015 PMC4385180

[btad556-B52] White JJ , Mazzeu JF, Hoischen A et al; Baylor-Hopkins Center for Mendelian Genomics. DVL3 alleles resulting in a -1 frameshift of the last exon mediate autosomal-dominant robinow syndrome. Am J Hum Genet 2016;98:553–61. 26924530 10.1016/j.ajhg.2016.01.005 PMC4800044

